# Supplementary material for: Evaluating the Anticholinergic Burden in Older Patients: Comprehensive Insights from a Nationwide Survey Among Emergency Medicine Specialists in the UK
Source: Geriatrics (Basel). 2025 Oct 24;10(6):137. doi: 10.3390/geriatrics10060137 (PMC12641666; doi:10.3390/geriatrics10060137)
Supplement: Supplementary file 1 [file geriatrics-10-00137-s001.zip › geriatrics-3905295-supplementary.pdf]

Supplementary S1: The ACB survey

☐ I understand that this questionnaire is fully anonymized and once I have submitted the completed form that I will be unable to withdraw from this study as all data will be fully anonymized.

Section 1

The demographics

1. Do you work in the Emergency Department?

☐ Yes

☐ No

2. What is your grade?

☐ Foundation Doctor

☐ Clinical fellow

☐ Core trainee

☐ Registrar

☐ Consultant

Section 2

Knowledge of the anticholinergic burden

This section tests how much you currently know about the anticholinergic burden. Please answer the questions below to the best of your own knowledge.

3. Are you aware of the term anticholinergic burden (ACB)?

☐ Yes

☐ No

4. If answered yes to the above, please choose a statement which best describe your understanding of the anticholinergic burden. (Multiple options can be selected)

☐ The result of taking multiple anticholinergic medications on the cognitive performance in the elderly.

☐ The cumulative effect of taking one or more medications with anticholinergic activity.

☐ Describes the proportion of medications with anticholinergic properties in the medication history of a patient with polypharmacy.

☐ Describes the number of co-morbidities associated with anticholinergic medications in a patient's past medical history.

5. Out of the 10 medications listed below, can you identify which have an anticholinergic burden score? (Please select one option for each row)

Medications	Yes	No	Don't know
Bisoprolol	☐	☐	☐
Loperamide	☐	☐	☐
Sertraline	☐	☐	☐
Furosemide	☐	☐	☐
Amlodipine	☐	☐	☐
Quinine sulphate	☐	☐	☐
Oxybutynin	☐	☐	☐
Diazepam	☐	☐	☐
Isosorbide mononitrate	☐	☐	☐
Metformin	☐	☐	☐

6. For the medications listed, can you quantify the anticholinergic burden score by anticholinergic cognitive burden scale? Please score the medications from 0 (no significant anticholinergic burden) to 3 (significant anticholinergic burden). (Please select one option for each row)

Medications	0	1	2	3	Don't know
Bisoprolol	☐	☐	☐	☐	☐
Loperamide	☐	☐	☐	☐	☐
Sertraline	☐	☐	☐	☐	☐
Furosemide	☐	☐	☐	☐	☐
Amlodipine	☐	☐	☐	☐	☐
Quinine sulphate	☐	☐	☐	☐	☐
Oxybutynin	☐	☐	☐	☐	☐
Diazepam	☐	☐	☐	☐	☐
Isosorbide mononitrate	☐	☐	☐	☐	☐
Metformin	☐	☐	☐	☐	☐

7. Which of the following short or long-term side effects do you think are associated with having a high anticholinergic burden score? (Please select one option for each row)

Side effects	Yes	No	Don't know
Falls	☐	☐	☐
Osteoporosis	☐	☐	☐
Stroke	☐	☐	☐
Delirium	☐	☐	☐
Sarcopenia	☐	☐	☐
Cardiovascular disease	☐	☐	☐
Urinary incontinence	☐	☐	☐
Gastro-oesophageal reflux disease	☐	☐	☐
Dementia	☐	☐	☐
Open-angle glaucoma	☐	☐	☐

8. If you have some knowledge relating to the anticholinergic burden, where did you acquire this?

☐ Undergraduate training

☐ Post graduate training

☐ I do not have any knowledge relating to the anticholinergic burden.

Section 3

Attitudes towards the anticholinergic burden

In this section we would like to know your opinion on the anticholinergic burden associated with the use of anticholinergic medication.

9. Do you believe the anticholinergic burden is an important issue amongst older patients?

☐ Yes

☐ No

10. Do you believe Emergency Department staff can play a role in managing the anticholinergic burden issues in older patients?

☐ Yes

☐ No

11. How important do you believe it is for Emergency Department staff to have an awareness of the consequences of prescribing anticholinergic medications to older people?

- ☐ Not important at all
- ☐ Low importance
- ☐ Neutral
- ☐ Important
- ☐ Very important

12. How important do you believe it is for Emergency Department staff to be able to assess and quantify the anticholinergic burden in patients?

- ☐ Not important at all
- ☐ Low importance
- ☐ Neutral
- ☐ Important
- ☐ Very important

Section 4

Clinical Practice and the anticholinergic burden

In this section we would like to know what role the anticholinergic burden plays in your clinical practice

13. How often do you document a patient's medication history in your current role?

- ☐ Never
- ☐ Rarely
- ☐ Sometimes
- ☐ Often
- ☐ Always

14. Where would you acquire the patient's medication history? (Multiple options can be selected)

- ☐ Patient
- ☐ GP records

- ☐ Patient hospital record
- ☐ Carers
- ☐ Ambulance staff
- ☐ Community notes

15.Do you prescribe anticholinergic medications in your current role?

- ☐ Yes
- ☐ No
- ☐ Don't know

16. If answered yes to the above, how often do you consider the anticholinergic burden and/or use the anticholinergic burden score?

- ☐ Never
- ☐ Rarely
- ☐ Sometimes
- ☐ Often
- ☐ Always

17.How often do you calculate the anticholinergic burden score when you document a patient's medication history

- ☐ Never
- ☐ Rarely
- ☐ Sometimes
- ☐ Often
- ☐ Always

18. If/ when you do the anticholinergic burden score and find it to be high, what action would you then take? (Multiple options can be selected)

- ☐ Inform GP
- ☐ Inform admitting clinical team
- ☐ Discuss with the patient

☐ Do nothing

☐ Others …………………………………………………. If answered "Other" option, please specify.

19. Do you believe that more education around the anticholinergic burden is required in the Emergency Department staff?

☐ Yes

☐ No

Survey feedback

We would like to know what you thought about the ACB survey. All feedback is valued and will help us improve the survey for future use.

20.Please rate your experience of completing the ACB survey.

Unsatisfactory

1

2

3

4

5

6

7

8

9

10

Excellent

21.Please use the space below to provide any additional comments or suggestions for us to improve the survey.

End of the survey

Thank you for taking the time to complete this survey, we look forward to hearing your thoughts.
